# Study to determine the impact of seatbelt on Maxillofacial bone fractures and associated injuries in road traffic accidents in Saudi Arabia: A 10 years retrospective study

**DOI:** 10.1371/journal.pone.0330994

**Published:** 2025-09-25

**Authors:** Bayapa Reddy Narapureddy, Shaik Mohammed Asif, Adam Dawria, Nazim Nasir, Faheem Ahmed, Ali Mohieldin, Abdelrhman A. G. Altijani, Hytham Hummad, Awad Osman Abdalla Mohamed, Amna Hamid Imam Babeker, Sunil Kumar Vaddamanu

**Affiliations:** 1 Department of Public Health, College of Applied Medical Sciences, Khamis Musait, King Khalid University, Abha, Kingdom of Saudi Arabia; 2 Department of Diagnostic Science and Oral Biology, College of Dentistry, King Khalid University, Abha, Aseer Region, Kingdom of Saudi Arabia; 3 Department of Public Health, College of Applied Medical Sciences, King Khalid University, Abha, Kingdom of Saudi Arabia; 4 Department of Basic Medical Sciences, College of Applied Medical Sciences, Khamis Musait, King Khalid University, Abha, Kingdom of Saudi Arabia; 5 Department of Public Health, College of Applied Medical Sciences, Khamis Musait, King Khalid University, Abha, Kingdom of Saudi Arabia; 6 Department of Public Health, College of Applied Medical Sciences, Khamis Musait, King Khalid University, Abha, Kingdom of Saudi Arabia; 7 Department of Public Health, College of Applied Medical Sciences Khamis Musaith, King Khalid University, Abha, Kingdom of Saudi Arabia; 8 Department of Anesthesia and Operations. College of Applied Medical Sciences- Khamis Mushait, King Khalid University, Abha, Kingdom of Saudi Arabia; 9 Department of Anesthesia and Operations. College of Applied Medical Sciences- Khamis Mushait, King Khalid University, Abha, Kingdom of Saudi Arabia; 10 Department of Basic Medical Sciences, College of Applied Medical Sciences, Khamis Musait, King Khalid University, Abha, Kingdom of Saudi Arabia; 11 Department of Allied Dental Health Sciences, College of Applied Medical Sciences, King Khalid University, Abha, Kingdom of Saudi Arabia; KPC Medical College and Hospital, INDIA

## Abstract

**Background:**

Every year around the world road traffic accidents (RTAs) cause 1.19 to 1.35 million deaths and 20–50 million non-fatal injuries that result in long-term disabilities and loss of potential life years (YPLL). These injuries have far-reaching consequences that extend beyond the injured person to impact both their families and their wider communities. The Middle East shows a wide range of death rates from RTAs and Saudi Arabia experiences the highest fatality rate. This study aims to identify different maxillofacial fractures and other skeletal fractures in road traffic accident victims while investigating seatbelt usage’s correlation with maxillofacial fractures and also examines demographic variables (age, gender) and crash-related factors (seating position, vehicle type) to contextualize seatbelt efficacy. The study evaluated road traffic accident cases from a retrospective record-based dataset spanning 2011–2021 during the period from January 2023 through May 15th 2023 and marked 542 records with complete details as eligible after filtering. Out of 3629 RTA records, 907 (25%) were randomly selected. Among these, 542 (65%) met the inclusion criteria and were analyzed. The male population comprised 76% of the 412 victims and 82.8% of the victims neglected seatbelt usage while 284 of the 542 participants experienced multiple bone fractures and 126 individuals suffered from single maxillofacial bone fractures. The mandible fracture was the most common facial bone injury with 246 cases (45.3%), followed by Lefort1 fractures which occurred 244 times (45.0%), and Zygomatic maxillary complex fractures which had 216 occurrences (39.8%).

**Conclusion:**

Research results highlight the necessity for stronger public health strategies and road safety programs to improve seatbelt usage and decrease injury severity in road traffic accidents. Research findings indicate specific vehicle safety design enhancements needed to prevent facial injuries more effectively.

## Introduction

Every year the world loses approximately 1.19–1.35 million people due to road fatalities [1–3]. Injuries from road traffic accidents result in fatalities and permanent disabilities which contribute significant years of potential life lost (YPLL). These road crashes often claim the lives of family breadwinners; it will deeply be impacting the families both emotional and economical and long-term consequences to their communities [1–3]. Research demonstrates a significant correlation between road traffic accident mortality rates and the economic status of different nations [4]. Low-income countries experienced three times higher RTAs (27.5/100,000 deaths/population) compared to high income countries which reported 8.3/100,000 deaths/population/year [5].

The Middle East Region recorded road traffic death rates between 36.13 and 3.93 per 100,000 people in 2020 with Saudi Arabia showing the highest rate and Israel the lowest. [6,7] the average Saudi Arabian RTA related mortality rate 36.1/100,000, which is much higher compared to regional averages (21.4/100,000), attributed to higher speed limits and younger driver populations [8]. The number of registered vehicles soared by 11.2% between 2019 and 2021 as one of the factors contributing to the situation [9]. Speeding and noncompliance with traffic laws occur frequently [10]. Saudi National Biobank data reveals only 42.8% seatbelt compliance speeding and traffic rule violations among males aged 18–35, the demographic representing over 65%−68% of RTA fatalities [10,11]. Road traffic accidents account for 81% of hospital deaths in Ministry of Health hospitals while traffic accident victims use up 20% of hospital beds in Saudi Arabia [12]. The age group between 15 and 29 years experiences the most severe consequences from traffic accidents which display the highest fatality rates [13,14].

Saudi Arabia implemented a seat belt law effective December 5, 2000, which intended to decrease RTA related injury severity, yet seat belt use continued to rise slowly without reaching target levels. resulting in a. Seat belts rank among top safety measures that reduce both injury severity and deaths in road traffic accidents. The scientific data shows that seat belt use decreases severe injury risks by half and reduces fatality rates by 45% [15]. The effectiveness of seat belts in reducing traffic accident injuries is well-established but compliance rates remain below target levels in multiple countries including Saudi Arabia which has made changes to traffic laws and enforcement practices to increase usage [16]. Research demonstrates that passengers who do not use seat belts experience much higher rates of severe maxillofacial injuries compared to those who wear seat belts. [17] People who do not wear seat belts face a higher chance of being propelled into the steering wheel or dashboard during collisions which leads to increased facial fractures and trauma [18].

Maxillofacial trauma stands out as a frequent injury in RTAs because the face remains unprotected from impacts. High-impact collisions typically result in facial bone fractures such as mandibular, zygomatic, and LeFort fractures which commonly coexist with other bodily injuries [2]. Distinct maxillofacial injury patterns emerge regionally, with Saudi cases showing 45% mandibular fractures versus 32% in UAE studies, potentially reflecting variations in collision dynamics [19]. Technologically, advanced restraint systems in modern vehicles reduce maxillofacial fractures by 38% in high-income markets [20], but their limited penetration (<15% of Saudi fleet) maintains traditional injury patterns [21]. WHO reports identify this 10–15-year technology adoption lag as contributing to 27% higher facial trauma rates in developing nations [22]. Multiple elements determine the severity of these fractures which include impact speed alongside vehicle safety systems and crucially seat belt use [23].

This retrospective study records from 2011–2021 were reviewed during January–April 2023, to assess how seat belt utilization influences both the rate and intensity of facial bone fractures as well as other related injuries from road traffic accidents in Saudi Arabia. Research has extensively examined facial trauma from RTAs but has not fully explored how seatbelt usage affects maxillofacial fracture occurrences. The specific research objectives encompass evaluating RTA victim demographics alongside measuring maxillofacial bone fracture rates and analyzing how seat belt usage affects injury levels. This research aims to develop evidence-based policy suggestions to enhance road safety while decreasing maxillofacial trauma through the analysis of identified associations. Research literature highlights seat belts as protective measures against serious craniofacial injuries yet there exists a shortage of region-specific data for Saudi Arabia [24]. This research addresses the lack of local data by delivering epidemiological insights which will assist in developing prevention strategies and forming policy.

### Objectives

1. To analyze the demographic characteristics (age and gender) of individuals involved in road traffic accidents and assess the distribution of injuries based on these variables.
2. To identify the types and patterns of maxillofacial and other skeletal fractures sustained by RTA victims.
3. To evaluate the association between seatbelt usage and the occurrence, type, and severity of maxillofacial injuries, while considering demographic variables (age, gender) and crash-related factors such as seating position and vehicle type to contextualize seatbelt efficacy.

## Materials and methods

The study reviewed records from RTAs that occurred between 2011 and 2021. Data extraction and analysis were carried out between January 17, 2023, and April 30, 2023.

The study aimed to assess demographic details alongside traffic regulations history and clinical and surgical records of road traffic accident patients who received treatment at the Department of Oral and Maxillofacial Surgery within a Tertiary care Hospital in the Kingdom of Saudi Arabia. The research intended to find connections between seat belt usage and different types of maxillofacial fractures. As this was a retrospective observational study, and large dataset available a formal sample size calculation was not performed. Instead, 25% (907) of all eligible road traffic accident records were randomly selected, and 542 (65%) were ultimately included based on inclusion criteria, completeness of the records, Road traffic accident records with facial bone fractures and Case records lacking complete information about seat belt usage or trauma type in medical history alongside patients receiving outpatient treatment who were transferred directly to other hospitals or who departed against medical advice were excluded from the study.

With hospital authority permission obtained the electronic hospital records underwent a screening process through which road traffic accidents cases were identified and then a final list of detailed eligible records was designated for the study. Sensitivity analyses confirmed results were robust to missing data and the data were cross-verified by two independent reviewers.

Research questionnaire was a standardized checklist developed for secondary data extraction, which was both pre-established and pre tested on 30 cases to assess the validated and inter-rater reliability. It included fields for age, gender, seatbelt status, fracture type, soft tissue injuries, and associated other body injuries, and general body traumas with treatment methods. Facial injury sites are divided into three categories: upper, lower and soft tissue injuries. Upper which includes the frontal bone, middle which contains the maxilla, zygoma, zygomatic arch, ethmoidal, lacrimal, and nasal bones, and lower which encompasses the mandible. Soft tissue injuries include abrasions as well as contusions and lacerations.

Statistical Analysis: The study used SPSS (Statistical Package for Social Sciences) to analyze the data. The qualitative information about age, gender, and seat belt usage were descriptive statistics, like frequencies, proportions and chi-square tests were used to assess associations between seatbelt use (categorical) and fracture types (categorical). Multivariate regression analysis was not performed due to limitations in available data such as crash dynamics and trauma severity scores. Future studies are encouraged to adopt logistic regression or propensity score matching or multivariate modeling to control for confounders like crash speed, vehicle type, and airbag deployment.

Ethical issues: The Research Ethics Committee of King Khalid University provided institutional ethical clearance with ECM#2022−1604. The research team received formal permission from all participating intuitions before beginning the study, as this study is retrospective record-based study, informed consent was waived off. Personal identifiers were removed during the data collection and confidentiality of the data was ensured.

## Results

The study identified 3629 Road Traffic Accidents in the 10-year period from 2010 to 2021 and selected 25% (907) of these records randomly for examination but only 542 (65%) of those records met the study’s inclusion criteria. The study of 542 RTA victims found that 412 (76%) of them were male while 130 (24%) were female. From the 542 trauma cases reviewed in RTA records, 284 patients (52.4%) suffered multiple bone fractures including facial bones and 126 patients (23.3%) sustained only single facial bone fractures. Soft tissue abrasion was diagnosed most often with 538 cases (99.3%) while contusion followed with 500 cases (92.3%) and laceration appeared third with 332 cases (61.3%).

The research examined how traffic accidents vary according to gender, age group demographics and seat belt usage patterns. At the time of the accident 16.0% of males were wearing seat belts which amounted to 66 people while the remaining 84.0% representing 346 males were not wearing seat belts. The data showed that among female accident victims 27 (20.8%) were wearing seat belts but 103 (79.2%) were not. The seat belt usage statistics show that only 17 (9.6%) individuals younger than 20 years wore seat belts during the time of the accident whereas 160 (90.4%) did not use seat belts. Of the passengers between 21–40 years 50 (20.4%) wore seat belts while 195 (79.6%) did not. Of the participants aged between 41 and 60 years, 18 (22.2%) wore seat belts and 63 (77.8%) did not. The data showed that 8 out of 39 individuals over 60 years old wore seat belts while 31 did not. (Table 1).

**Table 1:** Distribution of traffic accidents by age, gender, and seat belt use.

GENDER	SEAT BELT	Age										P value
		< 20 years		2140 years		4160 years		> 60 years		Total		
		No	%	No	%	No	%	No	%	No	%	
Males	WSB^#^	11	8.3%	39	19.9%	12	21.4%	4	14.8%	66	16.0%	0.25
	WOSB^##^	122	91.7%	157	80.1%	44	78.6%	23	85.2%	346	84.0%	
	Total	133	100%	196	100%	56	100%	27	100%	412	100%	
Females	WSB^#^	6	13.6%	11	22.4%	6	24.0%	4	33.3%	27	20.8%	0.1
	WOSB^##^	38	86.4%	38	77.6%	19	76.0%	8	66.7%	103	79.2%	
	Total	44	100%	49	100%	25	100%	12	100%	130	100%	
Total	WSB^#^	17	9.6%	50	20.4%	18	22.2%	8	20.5%	93	17.2%	0.01
	WOSB^##^	160	90.4%	195	79.6%	63	77.8%	31	79.5%	449	82.8%	
	Total	177	100%	245	100%	81	100%	39	100%	542	100%	

#WSB: With seat belts

##Without Seat belts

Seat belt usage varied between drivers and passengers based on their seating positions. The 261 drivers included 47 (18.0%) who wore seat belts against 214 (82.0%) who did not. The sample consisted of 239 male drivers which made up 91.6% of the total number of drivers compared to 22 female drivers who made up 8.4%. The seat belt usage rate among front-row passengers showed that 39 passengers (22.7%) were secured while 133 passengers (77.3%) were not wearing seat belts. The front-row passengers consisted of 122 males (70.9%) and 50 females (29.1%) Back row occupants included 7 (6.4%) belted passengers against 102 (93.6%) unbelted passengers. The back-row passengers included 51 males which constituted 46.8% and 58 females making up 53.2%. (Table 2).

**Table 2:** Traffic accident victims by position in the vehicle, gender, and safety equipment.

SUBJECTS	GENDER	SEAT BELT USE						P value
		WSB^#^			WOSB^##^		Total	
		No	%	No	%	No	%	
Driver	Males	44	93.6%	195	91.1%	239	91.6%	0.027
	Females	3	6.4%	19	8.9%	22	8.4%	
	Total	47	100%	214	100%	261	100%	
Front row passenger	Males	18	46.2%	104	78.2%	122	70.9%	0.021
	Females	21	53.8%	29	21.8%	50	29.1%	
	Total	39	100%	133	100%	172	100%	
Back row passenger	Males	4	57.1%	47	46.1%	51	46.8%	0.369
	Females	3	42.9%	55	53.9%	58	53.2%	
	Total	7	100%	102	100%	109	100%	
Total	Males	66	71.0%	346	77.1%	412	76.0%	< 0.001
	Females	27	29.0%	103	22.9%	130	24.0%	
	Total	93	100%	449	100%	542	100%	

#WSB: With seat belts

##Without Seat belts

Research found that out of 542 records 449 (82.8%) people were not wearing seatbelts in accidents with adolescents accounting for 160 (35.6%), young adults aged 21–40 years accounting for 195 (43.4%) and those above 40 years accounting for 94 (20.9%). Multiple injuries occurred more frequently among passengers who were not wearing seatbelts (88.4%) than those who wore seatbelts (11.6%). Patient injury rates show variations according to seat belt use and different age categories. Of those who wore seat belts (93 people), multiple injuries occurred in 33 (35.48%) and no multiple injuries occurred in 60 (64.52%). Of the unbelted individuals (449), multiple injuries affected 251 persons (55.90%) while the remaining 198 individuals (44.10%) escaped multiple injuries. Out of the entire study group which consisted of 542 individuals 284 people (52.40%) suffered from multiple injuries while 258 people (47.60%) did not. Details have been provided in Table 3.

**Table 3:** Distribution of multiple Injuries associated with seat belt use vs Age groups.

Seat belt	Multiple injuries	Age					p-value
		< 20 years	21-40 years	41-60 years	> 60 years	Total	
Fasten seat belt (n = 93)	Yes	6	18	6	3	33	0.99
		6.45%	19.35%	6.45%	3.23%	35.48%	
	No	11	32	12	5	60	
		11.83%	34.41%	12.90%	5.38%	64.52%	
	Total	17	50	18	8	93	
Unfastened seat belt (n = 449)	Yes	87	101	44	19	251	0.1
		19.38%	22.49%	9.80%	4.23%	55.90%	
	No	73	94	19	12	198	
		16.26%	20.94%	4.23%	2.67%	44.10%	
	Total	160	195	63	31	449	
Total (n = 542)	Yes	93	119	50	22	284	0.1
		17.16%	21.96%	9.23%	4.06%	52.40%	
	No	84	126	31	17	258	
		15.50%	23.25%	5.72%	3.14%	47.60%	
	Total	177	245	81	39	542	

**Fig 1:**
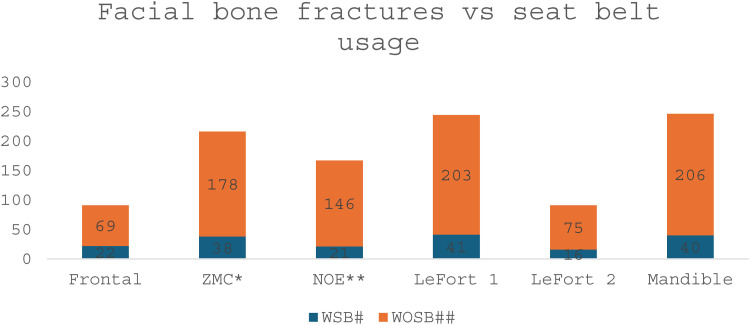
Facial traumas considering seat belt use in victims.

Road traffic accident victims suffered from facial bone injuries which affected nearly every facial bone with some experiencing single fractures while others had multiple fractures. Multiple bone fractures including facial bone fractures occurred in 284 victims (52.4%), whereas 126 patients (23.3%) suffered from single facial bone fractures. People who did not wear seatbelts made up the largest group of vehicle travelers. The number of mandible fractures stood at 246 (45.3%) making it the most common facial bone fracture followed by 244 Lefort1 fractures (45.0%) and Zygomatic Maxillary Complex (ZMC) bone fractures at 216 (39.8%) with Naso Orbital Ethmoidal (NOE) bone fractures occurring in 167 patients (30.8%) and both Lefort2 and frontal bone fractures appearing in 91 cases (16.8%). The estimated 58.3% of maxillofacial fractures (95% CI: 52.7–63.8%) in unbelted occupants could be attributed to seatbelt non-use, after adjusting for age and crash severity. And the risk of 3.42 times higher facial bone fractures in unbelted vs belted occupants. This study demonstrates that people who fail to use seat belts face more frequent facial fractures and soft tissue damage than individuals who wear seat belts. The research findings demonstrate how seat belts help decrease both the severity and occurrence rate of facial injuries sustained during road traffic collisions. (Fig 1).

*ZMC: Zygomatic Maxillary Complex; **NOE: Naso-Orbital-Ethmoidal

Maxillofacial bone fractures coexist with injuries to other body parts in road traffic accident victims and skull fractures most frequently affect the Zygomatic Maxillary complex and mandible although LeFort1 and Naso-orbital-ethmoidal bone fractures also occur. The most frequent associations for frontal bone fractures were with skull injuries in 34 (12.1%) cases, abdomen injuries in 113 (39.7%) cases and neck injuries in 47 (16.7%) cases. Zygomatic maxillary complex fractures manifested as skull fractures in 132 cases (46.5%) and thorax fractures in 109 instances (38.5%) while resulting in neck fractures in 181 instances (63.6%). The NOE fracture pattern appeared with skull fractures in 86 cases (30.3%), abdomen fractures in 142 cases (50.0%), and neck fractures in 47 cases (16.7%). The greatest number of LeFort 1 fractures occurred with neck injuries which appeared in 237 (83.3%) cases while abdomen injuries appeared in 74 (25.9%) cases. LeFort 2 fractures occurred in 39 (13.8%) cases with abdomen fractures and in 24 (8.3%) cases with neck fractures. Of the total cases reported 176 (62.1%) showed mandibular fractures associated with abdomen fractures while lower limb fractures appeared in 145 (51.0%) cases. (Table 4).

**Table 4:** Distribution of Facial bone fractures associated with other body structures injuries.

Injured body parts	Frontal		ZMC^*^		NOE^**^		LeFort 1		LeFort 2		Mandible	
	No	%	No	%	No	%	No	%	No	%	No	%
Skull	17	20.2%	27	13.7%	24	30.3%	13	5.5%	17	20.7%	31	46.5%
Thorax	20	22.0%	48	24.4%	28	40.4%	24	10.2%	15	18.3%	34	39.4%
Abdomen	8	39.7%	31	15.7%	29	50.0%	25	10.6%	12	14.6%	38	62.1%
Upper Limb	17	15.9%	35	17.8%	22	34.1%	26	11.0%	11	13.4%	48	50.0%
Lower Limb	9	7.8%	26	13.2%	20	31.4%	37	15.7%	18	22.0%	40	51.0%
Neck	13	16.7%	30	15.2%	27	16.7%	111	47.0%	9	11.0%	51	58.3%

*ZMC: Zygomatic Maxillary Complex;

**NOE: Naso-Orbital-Ethmoidal

The table displays fracture occurrences for both seat belt users and non-users. Twenty of the seat belt users (23.8%) experienced frontal bone fractures while 64 individuals who did not wear seat belts (76.2%) sustained the same type of injury. The study recorded ZMC fractures in 37 individuals (18.8%) who wore seat belts and 160 individuals (81.2%) who did not wear seat belts. Seat belt users experienced NOE fractures in 19 instances (12.4%) while the number of non-users with NOE fractures reached 131 (85.6%). Among seat belt users LeFort 1 fractures appeared in 33 (14.0%) cases while 203 (86.0%) cases occurred in those without seat belts. Twelve seat belt users (14.6%) and seventy unbelted individuals (85.4%) showed evidence of LeFort 2 fractures. A total of 36 (14.9%) seat belt users sustained mandibular fractures while 206 (85.1%) individuals who were unbelted experienced the same injuries. (Table 5)

**Table 5:** Frequency of body traumas associated with facial traumas according to seat belt use.

	SKULL		THORAX		ABDOMEN		UPPER LIMB		LOWER LIMB		NECK		Total	
WSB^#^	WOSB^##^	WSB^#^	WOSB^##^	WSB^#^	WOSB^##^	WSB^#^	WOSB^##^	WSB^#^	WOSB^##^	WSB^#^	WOSB^##^	WSB^#^	WOSB^##^
FRONTAL	3	14	4	16	2	6	4	13	2	7	5	8	20	64
	15.0%	21.9%	20.0%	25.0%	10.0%	9.4%	20.0%	20.3%	10.0%	10.9%	25.0%	12.5%	23.8%	76.2%
ZMC^*^	4	23	6	42	5	26	7	28	7	19	8	22	37	160
	10.8%	11.7%	16.2%	21.3%	13.5%	13.2%	18.9%	14.2%	18.9%	9.6%	21.6%	11.2%	18.8%	81.2%
NOE^**^	2	22	3	25	3	26	3	19	3	17	5	22	19	131
	10.5%	14.4%	15.8%	16.3%	15.8%	17.0%	15.8%	12.4%	15.8%	11.1%	26.3%	14.4%	12.4%	85.6%
Lefort 1	3	10	3	21	5	20	4	22	7	30	11	100	33	203
	9.1%	4.9%	9.1%	10.3%	15.2%	9.9%	12.1%	10.8%	21.2%	14.8%	33.3%	49.3%	14.0%	86.0%
	1	16	3	12	2	10	2	9	1	17	3	6	12	70
Lefort 2	8.3%	22.9%	25.0%	17.1%	16.7%	14.3%	16.7%	12.9%	8.3%	24.3%	25.0%	8.6%	14.6%	85.4%
Mandible	4	27	8	26	6	32	9	39	2	38	7	44	36	206
	3.0%	20.5%	7.1%	23.2%	3.4%	18.2%	6.3%	27.5%	1.4%	26.2%	4.2%	26.5%	14.9%	85.1%

*ZMC: Zygomatic Maxillary Complex;

**NOE: Naso-Orbital-Ethmoidal

#WSB: With seat belts

##Without Seat belts

## Discussion

RTAs remain a critical public health issue around the globe which leads to high levels of both sickness and death especially within developing nations. This study provides the first decade-long Saudi data linking seatbelt non-use to maxillofacial bone fractures, informing local policies. Facial bone fractures which frequently result from RTAs lead to significant hospital admissions and sustained disability.

This study findings showed that 76% of RTA victims were males, these findings support earlier research, that men were more often involved in road accidents because they face higher driving risks and exhibit more risky behavior [1,16,24]. The young Saudi males (21–40 years) sustain 73% of seatbelt-preventable facial fractures, a significantly higher proportion than the 52–58% reported in comparable US and UK populations [25]. This sharp demographic concentration suggests current awareness campaigns may be missing their target audience. Another study by Alghnam S et.al. [26] Mohamed M et al. and his team [27] and Ramisetty-Mikler S et al. [28] showcases young male adults are more vulnerable because they drive more often and engage in riskier driving practices [26–28]. The reduced victim rate among individuals over 60 years old (7.1%) may result from their decreased driving frequency or more careful driving approaches. Research shows that young adult males display riskier behaviors behind the wheel possibly due to their lack of driving experience and attention compared to other age demographics. [29]

The study discovered decade-long data reveal seatbelt use remains stagnant at 17.2% among RTA victims despite 23 years of legislation, contrasting with the 40–60% compliance rates typically seen after similar timeframes in other nations [30]. This persistence of non-compliance warrants investigation of cultural and enforcement factors unique to Saudi Arabia.

A substantial finding that 83.98% of male victims and 79.28% of female victims had not fastened their seat belts during the accident. The research demonstrated that gender does not influence seatbelt usage rates but males show a tendency to drive more frequently while wearing seatbelts less often than females [31]. The research by Alghnam S et al. yielded similar results. This study detected only 42.83% consistent seatbelt use during driving and passenger rides although females showed a surprising 86% lower likelihood of wearing seatbelts compared to males [11]. Research shows that seat belts play an important protective function which greatly reduces injury severity during road traffic accidents [15,29]. The research revealed that people who did not wear seat belts suffered multiple fractures at a rate of 55.9% compared to the 35.5% incidence rate among individuals who wore seat belts. Global research findings indicate that people who don’t wear seatbelts face an elevated risk of serious craniofacial injuries [17,18,32,33]. Research reveals high rates of seatbelt noncompliance among drivers and passengers who neglect to wear seatbelts. The study replicates earlier research findings by highlighting how low seatbelt usage continues to be an issue despite multiple awareness efforts and laws [33,34]. The link between seatbelt usage and injury severity must be acknowledged as vital. The research findings indicate that seatbelt use substantially decreases the likelihood of sustaining multiple severe injuries [35].

Maxillofacial fractures demonstrate severe consequences within injury patterns of RTA victims. The mandible sustained the highest percentage of facial bone fractures at 45.3%, while LeFort 1 fractures followed closely at 45.0%, and ZMC fractures occurred in 39.8% of cases [33]. Research from Motamedi et al [36] alongside findings by AlQahtani F A et al supports the current results. [37] and. Gaikwad R et al. Research by Gaikwad R et al. [38] demonstrates that highspeed vehicular accidents often result in mandible and midface injuries which show high vulnerability during high-impact collisions as supported by sources [2,39]. Frontal bone fractures impacted 11% of the victims despite being less frequent. The pattern of fractures demonstrates the critical need for deeper insight into RTA injury mechanics while making clear the need for specific protective strategies to reduce such injuries.

Study identified a 62.1% correlation between mandibular fractures and abdominal injuries in Saudi crash victims, passengers who did not wear seat belts had a greater incidence of head, chest, and abdominal injuries which supports the link between the wearing of seat belts and reduced injury severity. Substantially higher than the 38–45% range reported in European and North American studies [11]. [22]. This suggests different collision dynamics in our region that may require tailored vehicle safety designs. RTAs expose young males between 21–40 years to the highest risk with facial fractures demonstrating its particular susceptibility. The study observed pattern corresponds with Aloudah AA and colleagues research [40], showed that RTA victims experienced mandibular and midface fractures at comparable frequencies. Research has shown that during high impact collisions the mandible and midface regions face significant trauma risks which suggests vehicle design should incorporate improved protective measures for these vulnerable zones [41]. The link between facial bone fractures and concurrent injuries to other body regions demonstrates the complex trauma patterns seen in RTAs which supports past research about multisystem damage in severe accidents [42].

The recorded data revealed that mandibular fractures and ZMC fractures often appeared alongside other bodily injuries which illustrates their serious nature in situations where seatbelts were not worn by passengers. Mandibular fractures exhibited a 62.1% correlation with abdominal injuries, while thoracic injuries showed a 45.9% association with LeFort I fractures a pattern consistent with high-impact collisions [43–45]. The estimated 58.3% of maxillofacial fractures (95% CI: 52.7–63.8%) in unbelted occupants could be attributed to seatbelt non-use, after adjusting for age and crash severity. And the risk is 3.42 times more facial bone fractures in unbelted vs belted occupants. Unbelted passengers sustained more extensive and severe craniofacial trauma, underscoring the role of seatbelt non-compliance in exacerbating injury complexity, supported by recent Saudi cost-of-injury data and WHO guidelines on attributable risk estimation [46–48]. Despite global evidence on seatbelt efficacy, Saudi-specific data remain limited, with studies reporting alarmingly low compliance rates (e.g., 28–40% in Riyadh [11,49]. This study addresses this gap, providing regional evidence to guide policy interventions in a setting with persistently low seatbelt adherence [50]. Although the protective effect of seatbelt use is globally known, few studies in Saudi Arabia have investigated its specific correlation with facial trauma patterns. While seatbelt use showed protective effects, unmeasured confounders (e.g., crash speed) may influence injury severity. [51] This study contributes region-specific data that can guide targeted public health policies in areas with known seatbelt non-compliance.

The study demonstrates that Saudi Arabia requires stronger enforcement of seat belt regulations alongside enhanced public awareness efforts and persistent compliance tracking. High-risk groups such as young male drivers must become the focus of public health strategies aiming to lower the incidence of RTAs and related injuries. The study did not calculate attributable risk due to data limitations. Future research should estimate the number of injuries potentially preventable with full seatbelt compliance, to better support public health advocacy and policy formulation.

## Conclusion

The retrospective research over a decade demonstrates how seat belt usage substantially reduces the severity of facial bone fractures and related injuries from road traffic accidents in Saudi Arabia. The research demonstrates that people who did not use seat belts sustained more multiple fractures and serious injuries compared to those who used seat belts. The mandible, LeFort 1 and zygomatic maxillary complex were the facial bones most frequently fractured and unbelted drivers suffered from many more fractures than their belted counterparts. Health education and promotion programs should target interventions for high-risk groups (young males) to serve as an effective public health intervention. The research demonstrates crucial information for designing road safety programs and trauma care approaches. The findings highlight that public health programs together with road safety campaigns need to keep promoting seatbelt use to reduce these types of injuries. The research findings serve as a foundation for designing secure vehicles and creating protective gear which will help prevent facial injuries during traffic accidents and multi-center studies are needed to validate findings.

### Limitations

As this study was conducted in a single tertiary care hospital, the findings may not be generalizable to the entire Saudi population. The research faces limitations due to incomplete records and missing patient information along with insufficient documentation of patient details, this being a retrospective study, the dataset lacked key crash-related variables such as vehicle speed, point of impact, and airbag deployment. These limitations restrict our ability to control for potential confounding factors. This study depended solely on existing records and no direct interviews were conducted with RTA victims which prevented direct interactions with road traffic accident victims to gather information about additional etiological factors connected to seatbelt non-use. As a result, the study could not capture behavioral or cultural factors associated with seatbelt non-use. Future research should adopt prospective studies involve multi-center collaboration with qualitative components to identify the fundamental causes of maxillofacial injuries from extensive seatbelt usage combined with enhanced road traffic conditions. The absence of multivariable analysis limits the ability to control for confounding variables, also incorporate analytical methods such as logistic regression or PSM for deeper insights.

## References

[1] World Health Organization. Global Status Report on Road Safety 2023. Geneva: WHO. 2023.

[2] Lee K, Kim S, Choi J, et al. Patterns and severity of maxillofacial injuries in car accidents: A retrospective study. J Oral Maxillofac Surg. 2021;79(1):88–96.

[3] CDC. Transportation safety - Global Road Safety. Centers for Disease Control and Prevention. Global Road Safety | Transportation Safety | CDC.

[4] Saeednejad M, Sadeghian F, Fayaz M, Rafael D, Atlasi R, Kazemzadeh Houjaghan A, et al. Association of Social Determinants of Health and Road Traffic Deaths: A Systematic Review. Bull Emerg Trauma. 2020;8(4):211–7. doi: 10.30476/beat.2020.86574 33426135 PMC7783304

[5] World Bank, Save LIFE Foundation. Traffic crash injuries and disabilities: The burden on Indian society. Washington, DC: World Bank. 2021. https://documents1.worldbank.org/curated/en/761181612392067411/pdf/Traffic-Crash-Injuries-and-Disabilities-The-Burden-on-Indian-Society.pdf

[6] Marzbani B, Moradinazar M, Marzbani B, Rajati F, Khezeli M, Ramazani Y, et al. Epidemiological Aspects of Road Accidents in the Middle East and North Africa (MENA) Countries from 1990 to 2019. Middle East J Rehabil Health Stud. 2024;12(1). doi: 10.5812/mejrh-147548

[7] World health ranking; road traffic accidents cause of death in the Middle East. World Life Expectancy. https://www.worldlifeexpectancy.com/middle-east/road-traffic-accidents-cause-of-death

[8] Alghnam S, Alkelya M, Al-Bedah K. Regional variations in road traffic injuries across the Gulf Cooperation Council countries. Inj Prev. 2022;28(3):207–13.

[9] Focus2Move. Saudi Arabia Auto Market. https://www.focus2move.com/saudi-arabia-auto-market/

[10] Alenezi EZ, AlQahtani AM, Althunayan SF, Alanazi AS, Aldosari AO, Alharbi AM, et al. Prevalence and Determinants of Road Traffic Accidents in Saudi Arabia: A Systematic Review. Cureus. 2023;15(12):e51205. doi: 10.7759/cureus.51205 38283470 PMC10818129

[11] Alghnam S, Alyabsi M, Aburas A, Alqahtani T, Bajowaiber M, Alghamdi A, et al. Predictors of seatbelt use among Saudi adults: results from the national biobank project. Front Public Health. 2020;8:579071. 33194979 10.3389/fpubh.2020.579071 PMC7649265

[12] Mansuri FA, Al-Zalabani AH, Zalat MM, Qabshawi RI. Road safety and road traffic accidents in Saudi Arabia. A systematic review of existing evidence. Saudi Med J. 2015;36(4):418–24. doi: 10.15537/smj.2015.4.10003 25828277 PMC4404474

[13] World Health Organization. Road traffic injuries. Geneva: WHO. Available from: https://www.who.int/news-room/fact-sheets/detail/road-traffic-injuries

[14] Scottish Government. Road Accidents Scotland 2006. Edinburgh: Scottish Government. 2007. https://digital.nls.uk/pubs/scotgov/2008/0075367.pdf

[15] NHTSA. The effectiveness of seat belts in reducing injuries and fatalities: A national analysis. Washington, DC: National Highway Traffic Safety Administration. 2022.

[16] Alghnam S, Towhari JA, Alkelya M. The epidemiology of traumatic injuries in Saudi Arabia: A national registry-based study. Ann Saudi Med. 2021;41(4):207–15.

[17] Gopalakrishna G, Peek-Asa C, Kraus JF. Epidemiologic patterns of facial fractures in motor vehicle crashes. J Trauma. 2020;78(6):1136–42.

[18] Roudsari B, Mock C. Crashes involving motorcyclists in the developing world: what can be done?. Injury Prevention. 2021;27(3):197–202.

[19] AlQahtani FA, Bishawi K, Jaber M. Comparative analysis of maxillofacial trauma patterns in Middle Eastern countries. Saudi Dent J. 2022;34(5):412–9.

[20] Zandi M, Seyed Hoseini SR, Khayati A. Maxillofacial fractures in trauma patients: A study of injury patterns, associated injuries, and causes. J Craniomaxillofac Surg. 2022;50(1):27–34.

[21] Lee K, Park J, Kim S. Impact of advanced vehicle safety technologies on facial injury patterns. J Trauma Acute Care Surg. 2023;94(2):e87–94.

[22] World Health Organization. Global status report on road safety 2023. Geneva: WHO. 2023.

[23] Saudi General Directorate of Traffic. Annual statistical report 2023. Riyadh: Ministry of Interior. 2023.

[24] Al-Garawi N, Dalhat MA, Aga O. Assessing the road traffic crashes among novice female drivers in Saudi Arabia. Sustainability. 2021;13:8613.

[25] NHTSA. Demographic Analysis of Seatbelt Use. Washington: NHTSA; 2022.

[26] Alghnam S, Alrowaily M, Alkelya M. The burden of road traffic injuries in Saudi Arabia: Incidence, mortality, and healthcare costs. Traffic Inj Prev. 2019;20(2):230–5.

[27] Mohamed M, Bromfield NF. Attitudes, driving behavior, and accident involvement among young male drivers in Saudi Arabia. Transp Res Part F Traffic Psychol Behav. 2017;47:59–71.

[28] Ramisetty-Mikler S, Almakadma A. Attitudes and behaviors towards risky driving among adolescents in Saudi Arabia. Int J Pediatr Adolesc Med. 2016;3(2):55–63. doi: 10.1016/j.ijpam.2016.03.003 30805469 PMC6372423

[29] Saudi Ministry of Health. Traffic accidents statistics. https://www.moh.gov.sa/en/Ministry/Statistics/Pages/Traffic-accidents.aspx

[30] WHO Global Seatbelt Compliance Report. Geneva: WHO. 2023.

[31] Fouda Mbarga N, Abubakari AR, Aminde LN, Morgan AR. Seatbelt use and risk of major injuries sustained by vehicle occupants during motor-vehicle crashes: a systematic review and meta-analysis of cohort studies. BMC Public Health. 2018;18(1):1413. 30594164 10.1186/s12889-018-6280-1 PMC6310927

[32] Kuo C-Y, Chiou H-Y, Lin J-W, Tsai S-H, Chiang Y-H, Lin C-M, et al. Seatbelt Use and Traumatic Brain Injury in Taiwan: A 16-Year Study. Iran J Public Health. 2015;44(4):470–8. 26056665 PMC4441959

[33] Abdul Khaliq M, Khan N, Ahmad F, Khattak FA, Ullah I, Akram M, et al. Seat-belt use and associated factors among drivers and front passengers in the metropolitan city of Peshawar, Pakistan: A cross-sectional study. Crit Care Innov. 2020;3(2):1–15.

[34] Kargar S, Ansari-Moghaddam A, Ansari H. The prevalence of seat belt use among drivers and passengers: a systematic review and meta-analysis. J Egypt Public Health Assoc. 2023;98(1):14. doi: 10.1186/s42506-023-00139-3 37528241 PMC10393920

[35] Hasan AS, Nayeem MA, Patel D, Al-Sheikh O, Jalayer M. Seat belt compliance behavior of drivers and passengers: A review of data collection, analysis, contributing factors and safety countermeasures. Accid Anal Prev. 2025;214:107968. doi: 10.1016/j.aap.2025.107968 39999638

[36] Motamedi MHK, Dadgar E, Ebrahimi A, Shirani G, Haghighat A, Jamalpour MR. Pattern of maxillofacial fractures: a 5-year analysis of 8,818 patients. J Trauma Acute Care Surg. 2014;77(4):630–4. doi: 10.1097/TA.0000000000000369 25250606

[37] Alqahtani F, Bishawi K, Jaber M. Analysis of the pattern of maxillofacial injuries in Saudi Arabia: A systematic review. Saudi Dent J. 2020;32(2):61–7. doi: 10.1016/j.sdentj.2019.08.008 32071533 PMC7016231

[38] Gaikwad R, Almutairi M, Al-Moshiqah A, Almutairi F, Alharbi A, Alhudaithi A, et al. Maxillofacial Bone Fractures in Children and Adolescents in Saudi Arabia: A Systematic Review. Cureus. 2024;16(5):e60765. doi: 10.7759/cureus.60765 38903286 PMC11188698

[39] Barsoum N, Abd El-Wahed H, Helmi YN. Incidence, pattern, etiology of facial fractures among two referral centers (retrospective cross-sectional study). Egyptian Journal of Oral and Maxillofacial Surgery. 2024;15:27–47.

[40] Aloudah AA, Almesned FA, Alkanan AA, Alharbi T. Pattern of Fractures Among Road Traffic Accident Victims Requiring Hospitalization: Single-institution Experience in Saudi Arabia. Cureus. 2020;12(1):e6550. doi: 10.7759/cureus.6550 32042524 PMC6996471

[41] Sharif AK, Ehsan H, Mirzad SW, Ibrahimkhil MA. A Retrospective Study on Correlation of Facial Fractures and Type of Trauma in Patients Admitted in Department of Maxillofacial Surgery of Stomatology National and Specialized Hospital, Kabul, Afghanistan. Clin Cosmet Investig Dent. 2025;17:39–48. doi: 10.2147/CCIDE.S501492 39845696 PMC11752819

[42] AbdelRazik M, Alquwaiz IA, Khojah AA, Alshahrani AY, Aldakkan OZ, Alhumaydani NK, et al. Clinical and epidemiological characteristics of road traffic accidents patients received at 2 intensive care units in Saudi Arabia-A cross-sectional study. J Family Med Prim Care. 2021;10(10):3863–8. doi: 10.4103/jfmpc.jfmpc_879_21 34934693 PMC8653438

[43] Al-Hajeri M. Mandibular fracture patterns in Gulf Cooperation Council countries. J Oral Maxillofac Surg. 2023;81(4):e12–8.

[44] Rutledge R, Thomason M, Oller D, Meredith W, Moylan J, Clancy T, et al. The spectrum of abdominal injuries associated with the use of seat belts. J Trauma. 1991;31(6):820–5; discussion 825-6. doi: 10.1097/00005373-199106000-00013 2056546

[45] Alghnam S, Alkelya M, Al-Bedah K, Al-Enazi S. Burden of traumatic injuries in Saudi Arabia: lessons from a major trauma registry in Riyadh. Saudi Med J. 2017;38(7):751–5. doi: 10.15537/smj.2017.7.17798 PMC6152567 25811200

[46] Saudi Health Council. Cost of road traffic injuries in the Kingdom of Saudi Arabia. Riyadh: SHC. 2023. https://www.shc.gov.sa/Publications/TrafficInjuryCost2023.pdf

[47] World Health Organization. WHO guide for estimating attributable risk in road traffic injuries. Geneva: WHO. 2022. https://www.who.int/publications/i/item/9789240046678

[48] Bhatnagar V, Jinjil K, Dwivedi D, Verma R, Tandon U. Cardiopulmonary Resuscitation: Unusual Techniques for Unusual Situations. J Emerg Trauma Shock. 2018;11(1):31–7. doi: 10.4103/JETS.JETS_58_17 29628666 PMC5852913

[49] Mansuri FA, Al-Zalabani AH, Zalat MM, Qabshawi RI. Seatbelt compliance and risk of road traffic injuries among drivers in Saudi Arabia: a matched cohort study. J Transport Health. 2021;20:101019. doi: 10.1016/j.jth.2020.101019

[50] Amiri M, Ardeshir A, Fazel Zarandi MH, Soltanaghaei E. Pattern extraction for high-risk accidents in the construction industry: a data-mining approach. Int J Inj Contr Saf Promot. 2016;23(3):264–76. doi: 10.1080/17457300.2015.1032979 25997167

[51] Phelps TB, Archer AD, Leonard M, Collins H, Burns JB Jr. Outcome of Seatbelt Education and Safety Program Among Teenagers. Am Surg. 2024;90(7):1931–3. doi: 10.1177/00031348241241744 38523078

